# Exploration on cold adaptation of Antarctic lichen via detection of positive selection genes

**DOI:** 10.1186/s43008-024-00160-x

**Published:** 2024-09-09

**Authors:** Yanyan Wang, Yaran Zhang, Rong Li, Ben Qian, Xin Du, Xuyun Qiu, Mengmeng Chen, Guohui Shi, Jiangchun Wei, Xin-Li Wei, Qi Wu

**Affiliations:** 1 State Key Laboratory of Mycology, Institute of Microbiology, Chinese Academy of Sciences, Beijing, 100101 China; 2 University of Chinese Academy of Sciences, Beijing, 100049 China; 3 School of Life Sciences, Yunnan University, Kunming, 650500 China; 4 Key Laboratory of Animal Ecology and Conservation Biology, Institute of Zoology, Chinese Academy of Sciences, Beijing, 100101 China

**Keywords:** Lichen-forming fungi, Polar region, Genome, Genomic syntenic alignment, Protein interaction network, G-protein signaling

## Abstract

Lichen as mutualistic symbiosis is the dominant organism in various extreme terrestrial environment on Earth, however, the mechanisms of their adaptation to extreme habitats have not been fully elucidated. In this study, we chose the Antarctic dominant lichen species *Usnea aurantiacoatra* to generate a high-quality genome, carried out phylogenetic analysis using maximum likelihood and identify genes under positive selection. We performed functional enrichment analysis on the positively selected genes (PSGs) and found that most of the PSGs focused on transmembrane transporter activity and vacuole components. This suggest that the genes related to energy storage and transport in Antarctic *U. aurantiacoatra* were affected by environmental pressure. Inside of the 86 PSGs screened, two protein interaction networks were identified, which were RNA helicase related proteins and regulator of G-protein signaling related proteins. The regulator of the G-protein signaling gene (*UaRGS1*) was chosen to perform further verification by the lichen genetic manipulation system *Umbilicaria muhlenbergii*. Given that the absence of UmRgs1 resulted in elevated lethality to cold shock, the role for UaRgs1 in Antarctic *U. aurantiacoatra* resistance to cold can be inferred. The investigation of lichen adaptation to extreme environments at the molecular level will be opened up.

**Supplementary Information:**

The online version contains supplementary material available at 10.1186/s43008-024-00160-x.

## INTRODUCTION

**Fig. 1:**
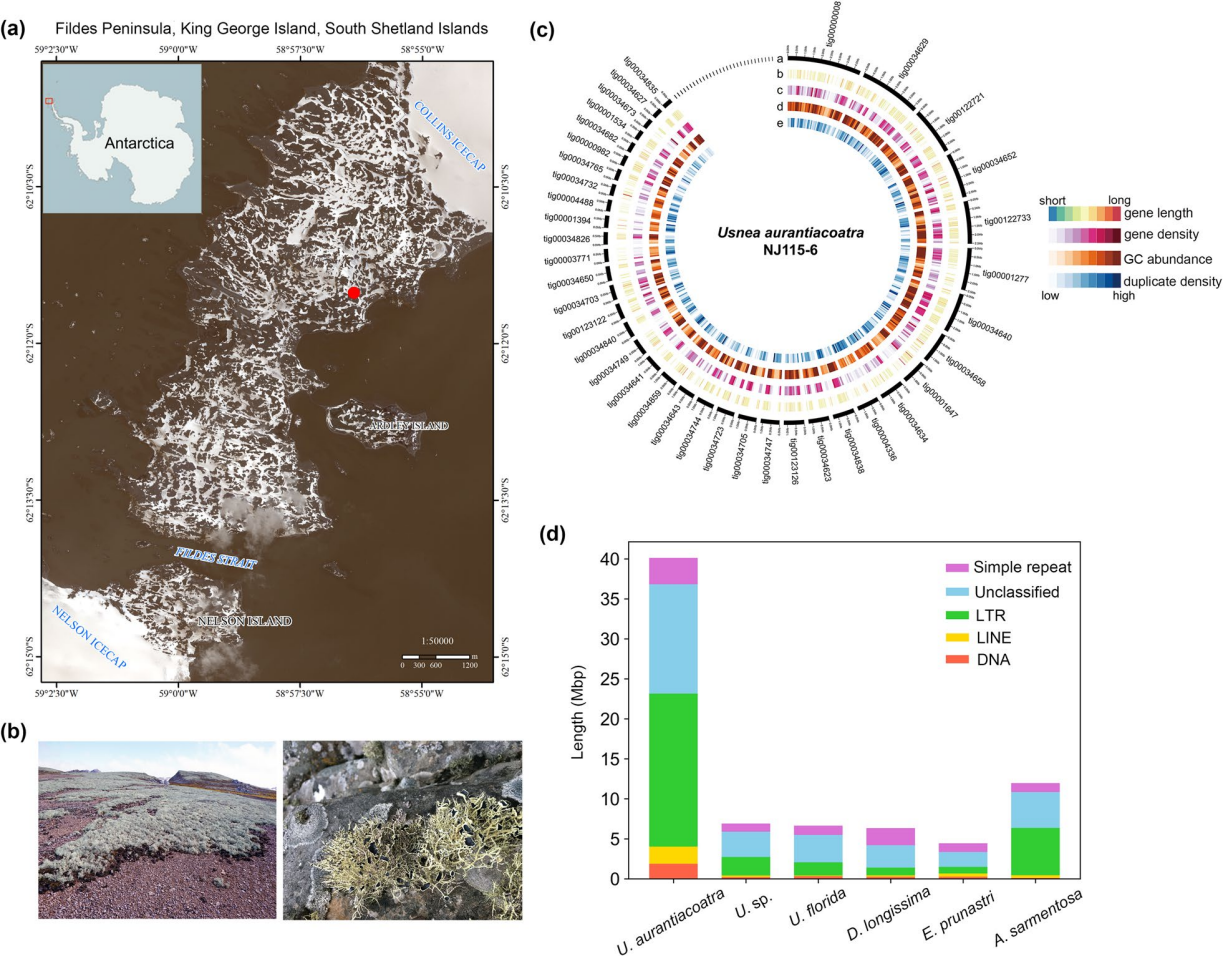
Antarctic *Usnea aurantiacoatra* and its genome annotations. **a** Location of the Fildes Peninsula on the Antarctic continent and the sampling site of *U. aurantiacoatra*. The sampling site is marked with solid red circles. **b** Habitat of *U. aurantiacoatra* in Ardley Island, which looks like a grassland from a distance. And the morphology of saxicolous lichen thallus in the field. **c** Genome annotations of *U. aurantiacoatra* (NJ115-6). Circos representation of detailed information about the genome. a: Contigs over 500 Kb are labeled with names; b: Gene length; c: gene density (gene numbers per 100 Kb); d: GC abundance (GC percentage per 100 Kb); e: duplicate density (duplicate numbers per 100 Kb). **d** Abundance of repetitive elements in six lichen genomes. The repetitive elements in *U. aurantiacoatra* was nearly 40 Mbp, which far exceeds the amount in the other five genomes

Lichens are stable mutualistic symbiosis composed of fungi and algae or cyanobacteria, also including a diverse microbiome (Honegger 1991, Lücking and Nelsen 2018, Hawksworth and Grube 2020; Zhang et al. 2023). In lichens, lichen-forming fungi (LFF) provide protection to algae, whereas the algae provide photosynthetic nutrition for the LFF. This symbiotic form allows lichens to be widely distributed in extreme environments worldwide, including polar, plateau, and desert regions. For this reason, lichens are known as “pioneer organisms” of the terrestrial ecosystem and stress-tolerant extremophiles (Yang et al. 2023). Taking the Antarctica as an example, it is the coldest continent in the world with 99.8% of the area covered by ice (Burton-Johnson et al. 2016). In its northern region, there are some ice-free and relatively mild sites called the Maritime Antarctica such as Fildes Peninsula of King George Island (Fig. 1a), where is characterized by annual mean temperature − 3 to − 4 °C, sometimes lower than − 10 to − 12 °C in over 8-month-long winter, and common freeze–thaw cycle. However, lichens not only can survive but also are predominant organisms with over 400 species in Antarctic vegetation here, as comparison, only two vascular plant species exist (Øvstedal and Smith 2001).

Although knowledge of the lichens has been accumulated on biodiversity and conservation (Romeike et al. 2002; Wauchope et al. 2019), as well as how they are influenced by Antarctic climate change (Sancho et al. 2017), the mechanism by which lichen species are adaptive to such an extreme environment and dominate the terrestrial vegetation is yet to be fully elucidated. The corresponding researches were only reported on animals (Li et al. 2014a; Daane and Detrich 2022) and green algae (Zhang et al. 2020) here. To our knowledge, no Antarctic lichen genome has yet been sequenced and analyzed. Moreover, the number of sequenced genomes is still too little in relation to the great variety of lichen species. The lack of enough genetic information, as well as limitations such as slow growth and difficulty in genetic manipulation, have slowed down the development of research on lichen resistance mechanism. In this context, genomics will be a more effective and feasible strategy.

Through our field investigation in the Fildes Peninsula of King George Island in Antarctica, we found the lichen species *Usnea aurantiacoatra* thriving there, and covering the ground gravel like grassland (Fig. 1a-1b), suggesting strong adaptation to the polar environment. Biogeographic and phylogenetic studies also indicated some special characteristics of this lichen. Firstly, majority of usneoid lichens distributed in low- and medium-latitude regions, including most species of *Usnea* and other related genera, whereas only several *Usnea* species including *U. aurantiacoatra* are confined in Antarctica. Secondly, these *Usnea* species confined in Antarctica and high-latitude represent one separate subclade, suggesting a significant genetic differentiation from the usneoid ancestors (Øvstedal and Smith 2001; Wirtz et al. 2006; Thell et al. 2012; Divakar et al. 2017). Based on these comprehensive characteristics, *U. aurantiacoatra* was chosen as our research model for cold adaptation of lichens.

In this study, three LFFs were de novo whole-genome sequenced, yielding a high-quality genome of *U. aurantiacoatra*. Genomic syntenic alignment was comprehensively performed in six usneoid LFFs, yielding a list of positively selected genes for environmental adaptation in *U. aurantiacoatra*. We performed functional enrichment and protein interaction network analysis and found that positively selected genes cluster into vacuole components, transporter proteins, RNA helicase, and G-protein signaling. We chose to use *Umbilicaria muhlenbergii* for functional validation of positively selected genes because *U. muhlenbergii* has genetic manipulation system (Wang et al. 2020, 2023), and it also grows extensively in cold region like *U. aurantiacoatra*, both of which belong to Lecanoromycetes. Protein interaction network analyses indicated that *UaRGS1* may play an important role in the adaptation of *U. aurantiacoatra* to the Antarctic environment, and that deletion of its ortholog, *UmRGS1*, in *U. muhlenbergii* resulted in the inability of *U. muhlenbergii* to tolerate cold-shock. Our results provided evidence for understanding the adaptation of *U. aurantiacoatra* to Antarctic environments and would open up the study of adaptive mechanisms in lichens at molecular level.

## METHODS

### Sample information

The *Usnea aurantiacoatra* lichen sample (NJ115-6) in minor amount was collected from the Fildes Peninsula, King George Island in Antarctica (Fig. 1a-1b, Table S1). The other two usneoid lichens, *Dolichousnea longissima* (coll.no. SC-9, syn. *Usnea longissima*) and an unknown *Usnea* species (coll.no. SC-4), were collected from the Sichuan Province of China (Table S1), considering their cold habitat source due to the high altitudes, and close phylogeny to *U. aurantiacoatra*. All samples were identified based on morphological observation and nuclear ribosomal DNA internal transcribed spacer (ITS) sequences, which were amplified using ITS4/ITS5 (White et al. 1990), and the sequencing results were blasted in the NCBI database.

### DNA extraction

The thallus of *U. aurantiacoatra* (NJ115-6), *D. longissima* (SC-9), and *U.* sp. (SC-4) were surface-cleaned and sterilized by immersion in 75% ethanol for 1 min, 1% sodium hypochlorite for 1 min, and sterile water for 1 min. Samples were then aired on sterile filter paper for half a day in a clean bench. The *U. aurantiacoatra* (NJ115-6) sample was directly sent to Nextomics Biosciences in Wuhan, China, and the thallus of *D. longissima* (SC-9) and that of *U.* sp. (SC-4) were sent to Majorbio in Shanghai, China, for DNA extraction and genomic sequencing.

### De novo whole-genome sequencing

A sequencing strategy, combining second-generation, Hi-C, and third-generation sequencing techniques, was used for the sequencing of usneoid lichen genomes. After quality checking, the qualified genomic DNA was used to construct a non-replication large-fragment genomic library with ~ 20 Kbp fragment sizes. The pooled library was bound to a polymerase and loaded onto a PacBio Sequel, after which PacBio Sequel SMRT Sequencing was performed. Hi-C libraries were prepared with independently extracted genomic DNA of the *U. aurantiacoatra* sample, and an Illumina sequencing library was constructed with a mean fragment size of ~ 350 bp for further Illumina Hiseq X10 sequencing. Hi-C and Illumina Hiseq sequencing data were used for genome-assisted assembly. For genomic DNA from samples of *U.* sp. (SC-4) and *D. longissima* (SC-9), independent Illumina Hiseq2500 sequencing was performed with 300- and 380-bp sequencing libraries, respectively, which was used for further de novo genome assembly.

### Genome resources available in public databases

Genome information of *Evernia prunastri* (Meiser et al. 2017), *Usnea florida* (https://mycocosm.jgi.doe.gov/Usnflo1/Usnflo1.home.html), and *Alectoria sarmentosa* (Liu et al. 2019a) used for comparative genome analysis were download from JGI (Grigoriev et al. 2014) and NCBI database. Genome information of *Letharia lupina* (McKenzie et al. 2020), *Imshaugia aleurites* (PRJEB42325), *Gomphillus americanus* (PRJEB42325), *Alectoria fallacina* (PRJEB42325), *Bacidia gigantensis* (Allen et al. 2021), *Physcia stellaris* (Wilken et al. 2020), *Letharia columbiana* (McKenzie et al. 2020), and *Lasallia pustulata* (Merges et al. 2023) used for analyzing amino acid residues of positively selected genes were downloaded from NCBI database.

### Genome assembly and annotation

The initial genome assembly was performed using FALCON software (Chin et al. 2013). For Hi-C data, Bowtie2 (v.2.3.2) (Langmead and Salzberg 2012) and HiC-Pro (v.2.8.1) (Servant et al. 2015) were applied for mapping reads, identifying valid interaction paired reads, and clustering scaffolds, respectively. Contigs with depths less than 50 × coverage were filtered out to exclude low-coverage genome sequences particularly potential algal genome sequences. FCS-GX was used to reconfirm the low degree of contamination from algae genomes (Astashyn et al. 2024). The final assembly was polished using independently sequenced Illumina Hiseq raw reads.

For protein-coding gene prediction, Augustus (Stanke et al. 2006) and GeneID (Parra et al. 2000) were used for de novo annotation, whereas GeMoMa (Keilwagen et al. 2016) and Genewise (Birney et al. 2004) were used for homologous annotation. The EVidenceModeler method (Haas et al. 2008) was used to integrate the two results for genomes sequenced in this study, including *U. aurantiacoatra* (NJ115-6), *U.* sp*.* (SC-4) and *D. longissima* (SC-9). The Funannotate (v1.8.13) with *Usnea florida* as the training model was used for genomes of *Evernia prunastri* and *Alectoria sarmentosa*. For *D. longissima* (SC-9), ORFs with both a start codon and a stop codon were retained to exclude incomplete annotations caused by short scaffolds.

For repeat sequence annotation, RepeatMasker (v.4.1.2) (Chen 2004), RepeatModeler (v.2.0.2) (Flynn et al. 2020), RepeatProteinMasker (v.4.1.2) (Chen 2004), and TandemRepeatFinder (v.4.09) (Benson 1999) were used with the repeat sequence database constructed with the *U. aurantiacoatra* (NJ115-6) assembly itself.

For ncRNA annotation, Rfam (Griffiths-Jones et al. 2005), tRNAscan-SE (Lowe and Eddy 1997), and RNAmmer (Lagesen et al. 2007) were used. For protein coding gene annotation, the first approach was to query the whole protein sequence to Swissprot (Bairoch et al. 2005) and KEGG (Ogata et al. 1999) databases, whereas the second approach was to use InterProScan (Zdobnov and Apweiler 2001) to identify conserved protein domains and annotate gene function. A circos plot (Krzywinski et al. 2009) was used to visualize the genomic landscape of related annotations.

### Orthologous gene identifications by genome syntenic alignment

We performed genomic syntenic alignment for six lichen genomes, including *U. aurantiacoatra* (NJ115-6), *U.* sp. (SC-4), *U. florida* (ATCC18376), *D. longissima* (SC-9), *E. prunastri* (FR SP7-11), and *A. sarmentosa* (MAF-Lich 21536), using LASTZ (version 1.04.00) (Chen et al. 2019; Hecker and Hiller 2020). The genome of *U. florida* was used as the reference genome, and the other five genomes were aligned to it. The five pair-align results were combined to obtain a final multispecies alignment as a multiple alignment format file. Then, the corresponding *U. florida* annotated coding sequence was used as reference to retrieve orthologous DNA sequences from the multiple alignment format file. This dataset of orthologous sequences of the six species included 12,243 genes and was a start-up orthologue dataset for further analyses.

### Evolutionary genetic analyses for adaptive selection

For positive selection analyses using PAML (v4.8), two tree-setting strategies were considered. Focusing on *U. aurantiacoatra* as the foreground branch, strategy #1 used all five species as background branches, and strategy #2 excluded *D. longissima* from the five species, using only four species as background branches. The reason to consider using strategy #2 was because the obvious fast growth rate of *D. longissima* (1–3 cm per year) compared with *U. aurantiacoatra* (4.3–5.5 mm per year) (Keon 2009; Esseen et al. 1981; Jansson et al. 2009; Li et al. 2014b). The results of the two tree strategies were combined to a final result. For each strategy, the same “more than 20% of the alignment in a gap” screening criterion was implemented. The result dataset of the two strategies included 5674 and 6202 orthologous genes, respectively, and their concatenated set was 6226 genes.

Based on the reconstructed phylogenomic tree, positively selected genes (PSGs) were identified using the branch-site model of the codon evolution with model = 2 and Nssites = 2, whereas for the branch model, the parameters were the null model (model = 0) versus the alternative model (model = 2). Genes with omega value (dN/dS) larger than 1 and statistically significant between foreground and background branch were regarded as PSGs. In PAML, a chi-square test is used to exam the statistical significance of the omega larger than 1. Besides, an FDR test was performed to exclude potential false positive PSGs.

To further confirm the target PSGs and find its potential amino acid residues where positive evolution occurs, eight additional lichen species with available genome data from Lecanoromycetes were added, including *Letharia lupina* (McKenzie et al. 2020), *Imshaugia aleurites* (PRJEB42325), *Gomphillus americanus* (PRJEB42325), *Alectoria fallacina* (PRJEB42325), *Bacidia gigantensis* (Allen et al. 2021), *Physcia stellaris* (Wilken et al. 2020), *Letharia columbiana* (McKenzie et al. 2020), and *Lasallia pustulata* (Merges et al. 2023). Orthologous genes of the target PSGs were identified from the total 14 genomes by a reciprocal BLAST strategy of INPARANOID algorithm (Remm et al. 2001). The potential amino acid residues were aligned to check their evolution and possible substitution variations.

### Protein interaction network

Protein interaction network analyses were performed in EMBL’s STRING (http://string.embl.de). Four different fungal model species were implemented as background genomes for analyses, including *Aspergillus nidulans*, *Cryptococcus neoformans*, *Neurospora crassa*, and *Saccharomyces cerevisiae*. For analyses the PSGs in *U. aurantiacoatra*, a 0.40 default interaction score of medium confidence was used. Domain analyses were performed in the SMART online website (http://smart.embl.de/smart/), with PFAM and signal peptide items checked.

### Generation of Δ*Umrgs1* mutant for functional verification

The *UaRGS1* ortholog in *Umbilicaria muhlenbergii*, *UmRGS1*, was identified by searching the genome (unpublished data) using a reciprocal BLAST strategy (Remm et al. 2001). The split-marker approach was applied to knock out *UmRGS1* in *U. muhlenbergii*. Upstream and downstream flanking sequences of *UmRGS1* were amplified using primers 1F/2R and 3F/4R (Table S2), respectively. The resulting PCR fragments were ligated onto the *hph* cassette amplified from pCX63 (Zhao et al. 2005) with primers HT-F/HY-R and YG-F/HT-R. The final *UmRGS1* gene replacement fragments were transformed into protoplasts generated by Driselase (Sigma-Aldrich), as described previously (Wang et al. 2020). Hygromycin-resistant transformants were screened by using PCR with primers 5F/6R, 7F/HY-R, YG-F/8R, and H850/H852 (Fig. S1).

The lichen-forming fungus *U. muhlenbergii* strain (wild type) and *UmRGS1* gene knockout mutant strains (LR2, LR5, and LR8) were routinely cultivated on potato dextrose agar medium at 20 °C for 7 days. To detect the survival rate of ultra-low-temperature stress, 2 × 10^3^ cells of the *U. muhlenbergii* JL3 strain and Δ*Umrgs1* mutants were subjected to six freeze–thaw cycles of liquid nitrogen without any protective agent. Cold-shocked cells were recovered on PDA medium at 20 °C for 10 days. Each experiment was performed in triplicate to count the number of colonies on the recovered plates.

## RESULTS

### De novo genome assembly combined multi-sequencing technologies yield high-quality genome of *Usnea aurantiacoatra*

To obtain a high-quality reference genome, we sequenced Antarctica *U. aurantiacoatra* (NJ115-6) (Fig. 1a-1b) using three sequencing techniques of PacBio, HiSeq (Illumina) and Hi-C. First, we assembled the genome sequences with 61.01 Gbp PacBio data using FALCON, and obtained a draft genome with a size of 81.0 Mbp (187 contigs; contig N50 944.85 Kbp) after removing the potential contaminant and redundant sequences. Then, we corrected the contigs with 86.83 Gb HiSeq data using pilon (v.1.24) (Walker et al. 2014). Finally, we linked the corrected contigs to scaffolds with 73.71 Gb Hi-C data using Bowtie2 (v.2.3.2), HiC-Pro (v.2.8.1), and LACHESIS (Burton et al. 2013), and obtained a total of 81.4 Mb genome sequences (121 scaffolds; Scaffold N50 1.59 Mb; Fig. 1c; Fig. S2; Table 1).

**Table 1:** Genome assembly statistics of usneoid lichens sequenced in this study

	*U. aurantiacoatra* (NJ115-6)	*U.* sp. (SC-4)	*D. longissimi* (SC-9)
Total Bases (Gbp)	86.83	9.83	11.50
Assembly size (Mbp)	81.40	40.60	67.30
No. Scaffolds	121	8966	59,938
Scaffolds N50 (Kbp)	1590	13.90	1.80
BUSCO (%)	97.36	88.51	54.22
Duplicated BUSCO (%)	16.41	0.47	1.70
Predicted genes	13,534	13,191	12,475
Accession	GWHBJEF00000000	GSA: CRA007127	GSA: CRA007127

We also performed Hiseq sequencing of the genome of two other usneoid lichen *U.* sp. (SC-4) and *D. longissima* (SC-9). We sequenced the genomic DNA (9.83 Gb for SC-4; 11.5 Gb for SC-9), and assembled a total of 40.6 Mb of SC-4 (contig N50 10.5 Kbp; scaffold N50 13.9 Kbp) and 67.3 Mbp of SC-9 (contig N50 1.3 Kbp; scaffold N50 1.8 Kbp) genome sequences using SOAPdenovo2, respectively (Table 1). Gene annotation obtained 13,534 protein-coding genes for *U. aurantiacoatra*, 13,191 genes for *U.* sp., and 12,475 genes for *D. longissima* (Table 1).

To carry out a comparative genome analysis, genome of *Usnea florida* (ATCC18376), *Evernia prunastri* (FR SP7-11), and *Alectoria sarmentosa* (MAF-Lich 21536), which belong to the same family (Parmeliaceae) as the three usneoid lichens mentioned above, were selected from the published genomic data. Of these six genomes, *U. aurantiacoatra* genome was the largest, almost double the size of the LFF genomes that have been published so far. To uncover the reason for the highly expanded genome size of *U. aurantiacoatra*, repeat sequence annotation was performed in these six LFF genomes. Comparison showed that the repeat sequences of *U. aurantiacoatra* were significantly higher than those of the other five genomes, with the greatest expansion of long terminal repeat (LTR) and unknown repeat sequences (Fig. 1d). The total size of all types of repeat sequence was about 40 Mbp, the same as the genome sizes of other lichen species except *U. aurantiacoatra*. Considering similar gene number in the six genomes (Table 1), it can be concluded that the large genome size in *U. aurantiacoatra* is due to amplification of repeat sequences. We also performed BUSCO assessment of the three usneoid LFF genome data obtained in this study, *U. aurantiacoatra* has the more complete genome (Fig. S3).

### Genome signatures for adaptive selection of *U. aurantiacoatra*

**Fig. 2:**
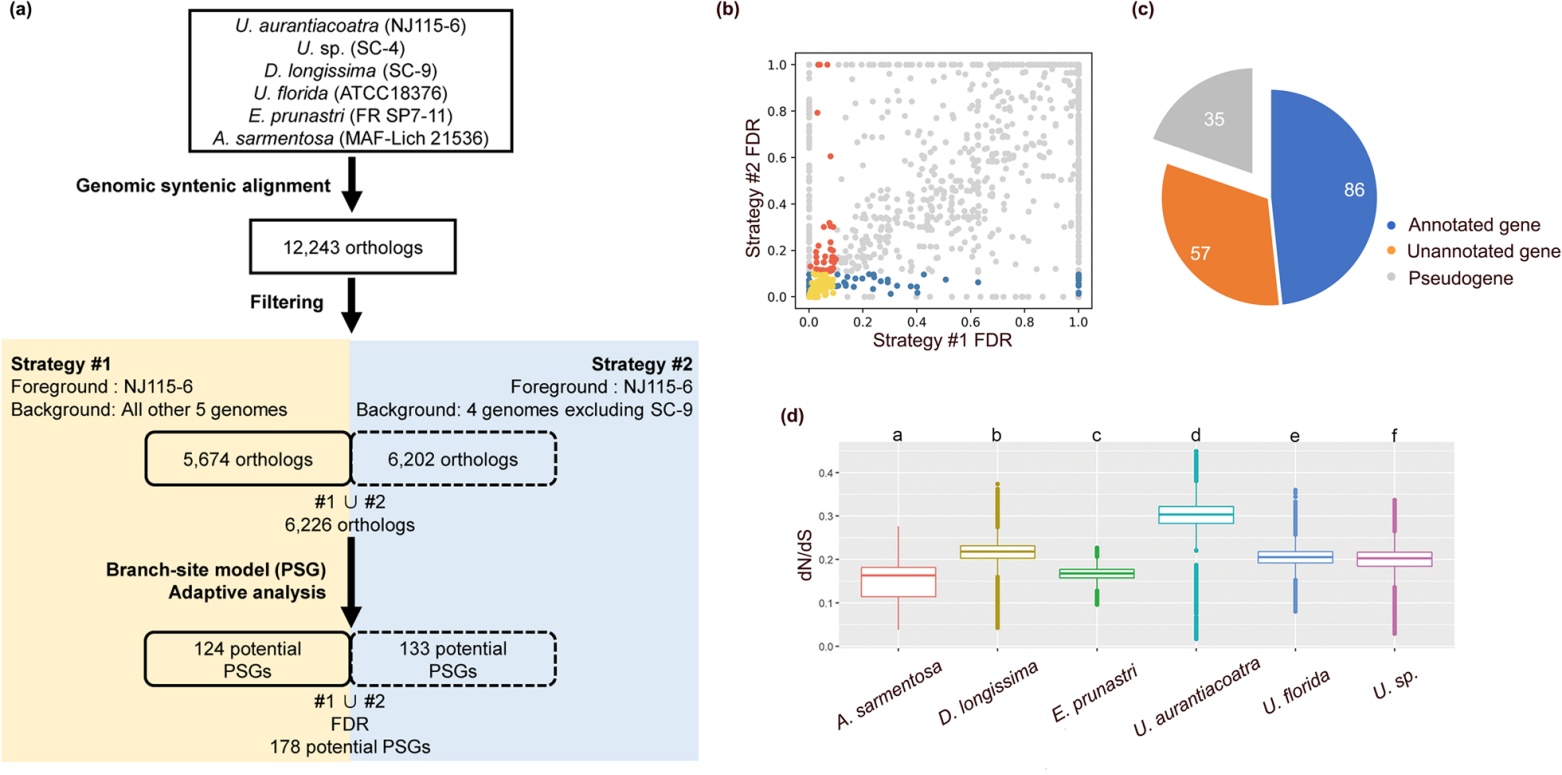
Positive selection related to the adaption to extreme Antarctic environments. **a** Workflow of evolutionary genetic analyses for adaptive selection. Genomic syntenic alignment found 12,243 orthologs in six genomes, which were filtered for two tree-building strategies. The concatenation obtained by both strategies was used for adaptive analysis, resulting in 178 potential positively selected genes (PSGs). **b** The *p*-values of the potential PSGs obtained by two strategies were corrected for false discovery rate (FDR) analysis. Red and blue dots indicated PSGs with *p*-values < 0.1 in strategy I and II, respectively. Yellow dots were PSGs with corrected *p*-values < 0.1 under both strategies, which were used for subsequent analysis. **c** Pie chart showing the number of pseudogenes, annotated genes, and non-annotated genes in 178 potential PSGs. **d** Box plot of mean dN/dS shows a significantly high mean ω value in *U. aurantiacoatra* (p < 0.01)

In search of adaptive selection traits at genomic level, 6226 concatenated genes obtained by two tree-setting strategies (Fig. 2a) were used for positive selection analysis by PAML, and were corrected the p-values with false discovery rate (FDR) analysis (Fig. 2b). There were 178 PSGs with corrected *p*-values < 0.1 under both strategies (Fig. 2b), removing 35 pseudogenes yielded 143 PSGs (Fig. 2c), which included 86 PSGs paired with genes annotated by EVidenceModeler (Table S3).

We calculated ω ratios (dN/dS) for 6226 orthologous genes, and comparisons revealed that the average value of the ω ratios for *U. aurantiacoatra* was significantly higher than that of other LFF (*p* < 0.01, Fig. 2d, Table S4–S5), indicating that negative selection in this species is universally more relaxed than other species.

### Functional enrichment and protein interaction network of positively selected genes

To acquire the biological processes and functions in which the above PSGs were involved, we performed functional enrichment analysis using clusterProfiler (Wu et al. 2021) with GO categories and KEGG pathways. By functional enrichment of 86 PSGs with a Q-value < 0.05, it showed that most of these proteins were associated with transmembrane transporter functions and vacuole components, and cyanoamino acid metabolism was also enriched (Table S6).

**Fig. 3:**
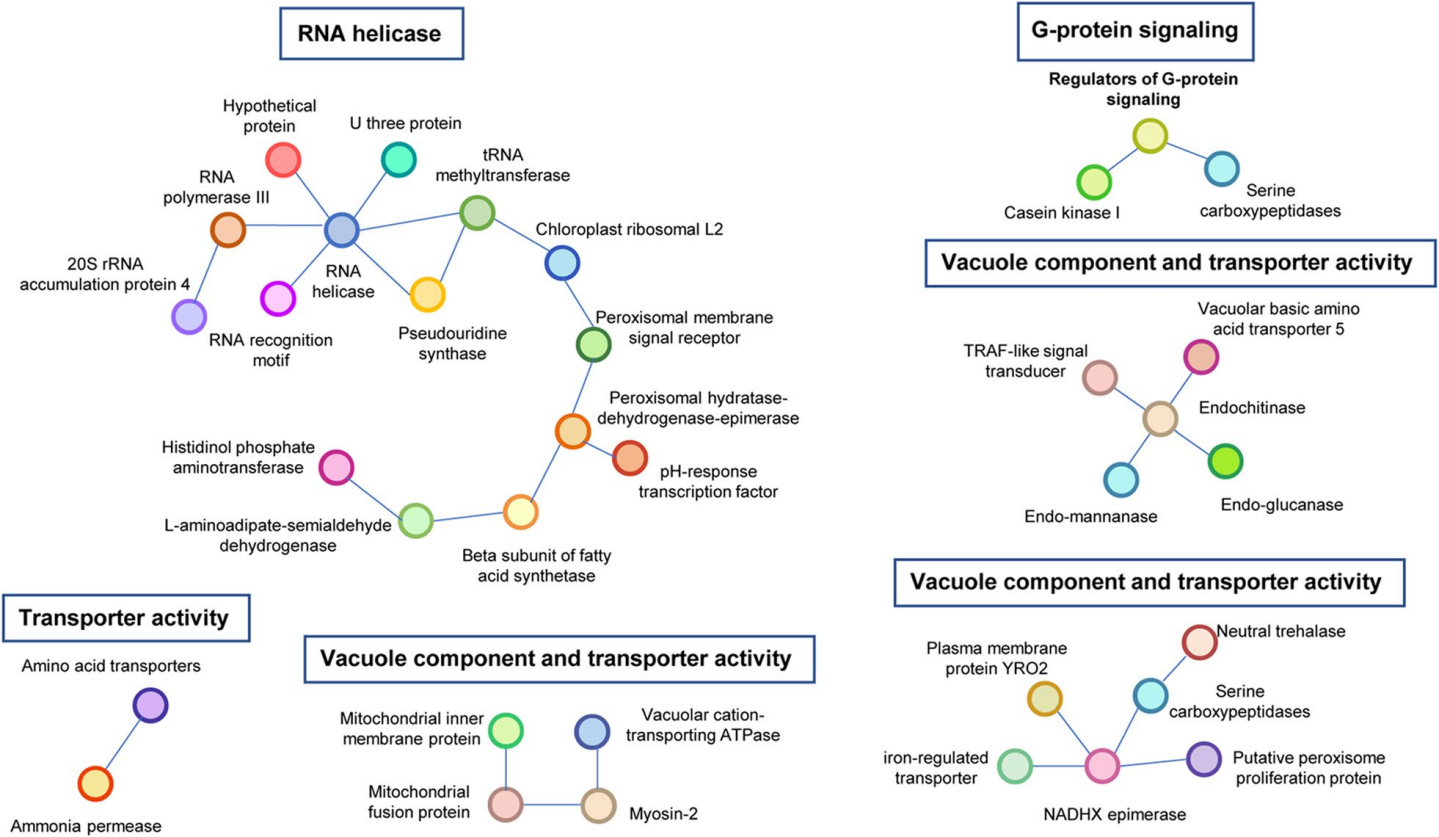
Protein interaction networks of positively selected genes (PSGs) in *Usnea aurantiacoatra*. A total of 35 PSGs were in six protein interaction networks, four of which were enriched for vacuole component and transmembrane transporter activity. In addition to this, a larger protein interaction network centered on RNA helicase, and another network of three proteins centered on regulator of G-protein signaling

To identify potential functional information related to adaptive selection, protein-associated network analysis was carried out with EMBL’s STRING platform using four model fungal species (*Aspergillus nidulans*, *Cryptococcus neoformans*, *Neurospora crassa*, *Saccharomyces cerevisiae*). Of the 4 model fungi species in STRING, we identified 6 protein interaction networks, which included 35 genes of the 86 PSGs (Table S7). PSGs in four of these protein interaction networks were enriched in the GO category described above, which were related to transmembrane transporter activity or/and vacuole component (Fig. 3). The other two protein interaction networks that were not enriched by KEGG or GO were the G-protein-signaling and RNA helicase (Fig. 3). The RNA helicase network involves 12 proteins with functions related to the processing and maturation of rRNA and tRNA modification in the initiation of protein translation (Fig. 3). The G-protein-signaling network contains three proteins, regulator of G-protein-signaling, casein kinase I, and carboxypeptidase (Fig. 3).

### Effects of regulator of G-protein-signaling in cold resistance

**Fig. 4:**
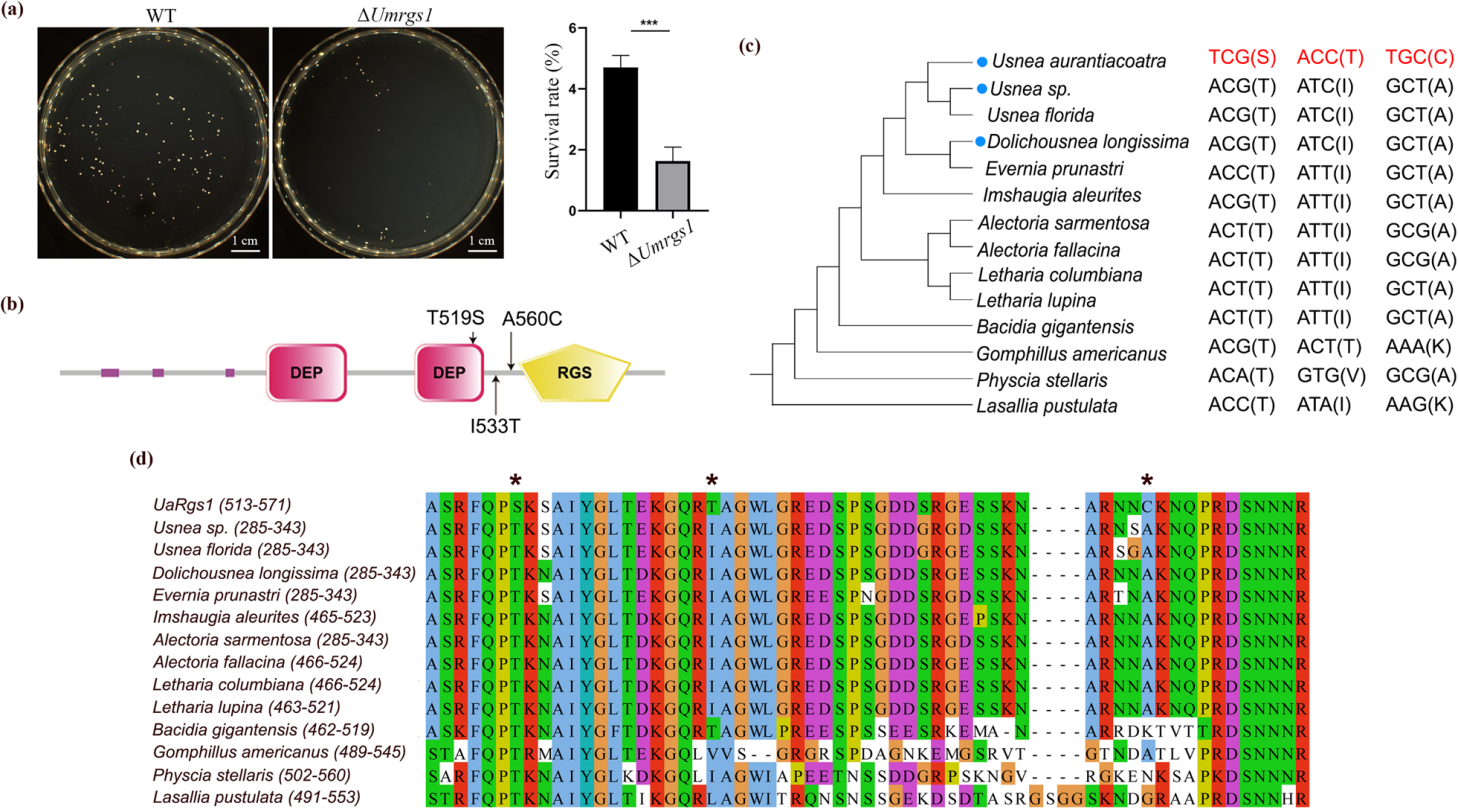
Functional verification of *UmRGS1* in rock tripe *Umbilicaria muhlenbergii*. **a** Survival colonies and survival rate of wild-type strain and Δ*Umrgs1* mutant after six liquid nitrogen freeze–thaw cycles. Error bars represent SD, unpaired T-test with Welch’s correction, *p* = 0.001, *n* = 3 independent experiments. **b** Domain architecture of UaRgs1 and the locations of three amino acid residues undergo positive evolution. **c** Phylogenetic tree constructed by UaRgs1 with its homologous in 13 other lichen-forming fungal genomes. List on the right were the amino acids and corresponding codons for the three positive selection sites in UaRgs1. **d** Multiple alignment of the same set of protein sequences, with asterisks marking the sites of three amino acids, and amino acid position 519 was in conserved domain

To characterize PSGs as evidence of environmental adaption in Antarctic *U. aurantiacoatra*, we selected regulator of G-protein-signaling to further study, and named this protein as UaRgs1. Since *U. aurantiacoatra* cannot be genetically manipulated, we chose to knock out the homologous of *UaRGS1* in *Umbilicaria muhlenbergii* (*UmRGS1*)*.* Three separate Δ*Umrgs1* deletion mutants (LR2, LR5, and LR8) were generated and validated by PCR using anchor primers (Fig. S1; Table S2). We chose LR2 as the representative of mutants for the further cold resistant verification considering the consistent phenotype among the three mutants. Knockout of the *UmRGS1* did not affect the growth phenotype of *U. muhlenbergii*. To determine whether UmRgs1 was critical for cold shock, the liquid nitrogen freeze–thaw experiment was performed on the wild-type strain and Δ*Umrgs1* mutants. After three parallel experiments, the average number of wild-type and Δ*Umrgs1* mutant colonies was 94 and 33 on the recovery medium, respectively (Fig. 4a; Table S8). The survival rate of wild-type strain (4.7%) was nearly three times that of the Δ*Umrgs1* mutant (1.65%), with a significant difference between them (*p* = 0.001), indicating that the Δ*Umrgs1* mutant is more sensitive to rapid freeze–thaw temperature changes than the wild-type. Therefore, we can deduce that UaRgs1 may have similar and even stronger function in Antarctic *U. aurantiacoatra* for resistance of the freeze–thaw sharp temperature changes.

We also identified potential amino acid residues where positive evolution occurs on UaRgs1 (Fig. 4b). A phylogenetic tree and multiple alignment using additional UaRgs1 orthologous in 13 LFFs, showed that amino acid 519 site, located in the DEP domain, was highly conserved but changed in Antarctic *U. aurantiacoatra* (Fig. 4c-4d), suggesting that the adaptive selection is associated with the biochemical and molecular function of the gene. Another branch site analysis for these 14 Rgs genes was conducted and the outcome showed that site 519 was under positive selection, which enhanced out conclusion.

## DISCUSSION

The three poles, Antarctica, Arctic and the Third Pole (i.e., the Tibetan Plateau and its surroundings), are the coldest environment on the Earth, of which Antarctica is the most severe one and recorded the lowest air temperature. Lichens, as the pioneer organisms, can dominantly survive in such extreme environments and contribute substantial biodiversity of their flora, for which a set of cold tolerance mechanisms are needed. Previous studies on the Antarctic lichens mainly focused on the correlation of lichen biodiversity and conservation with climate change (Sancho et al. 2017; Romeike et al. 2002; Wauchope et al. 2019). A related study revealed differences in biosynthetic gene clusters between *Umbilicaria pustulata* lichens in Mediterranean and cold-temperate regions (Singh et al. 2021). This suggests that there must be a large number of environmental adaptation genes in Antarctic lichen genome. In this case, *Usnea aurantiacoatra* was chosen as a model mainly due to our field investigation and the good matching between divergent time of this lichen and the paleoclimate and paleogeology of Antarctica discovered by our study, which indicated that it is very likely to have a strong adaption to the Antarctic extreme environment driven by natural selection.

The approaches identifying natural selection at the molecular evolution level involves estimation of synonymous and nonsynonymous substitution rates and detection of positive selection in protein-coding DNA sequences among species (Yang 2007). We comprehensively compared the genomes between *U. aurantiacoatra* and those lichens mainly living in the temperate regions, then attempted to remove *D. longissima* with different growth rates when analyzing PSGs. The results of the two strategies were slightly different (Fig. S4), but the results obtained from different background branches may be both meaningful. We used a combination (the union) of the two strategies to ensure that genes selected by either strategy would be included in the final set of result genes.

The identified 86 PSGs possibly related to cold adaptation mainly enriched in lytic and storage vacuole components. The fungal vacuole serves as both the main compartment for division and a reservoir for the storage of small molecules, such as polyphosphate, amino acids, divalent calcium, and other small molecules (Klionsky et al. 1990). In some fungi, genes controlling the formation of vacuole play an important role in resistance to stresses (Son et al. 2018; Wilken et al. 2020; Zhu et al. 2023). Antarctic lichens need to undergo long periods of dormancy, and it is hypothesized from our results that *U. aurantiacoatra* enhance its vacuole function under selective pressure.

Two other potentially important mechanisms were found through protein network analysis. RNA helicase at the center of the largest protein interaction network, has been intensively studied in both eukaryotes (O’Day et al. 1996) and bacteria (Liu et al. 2021). This gene involves in the maturation of 35S-pre-rRNA and is required for cleavages leading to mature 18S rRNA in cell nucleus, which plays a critical role for the biogenesis of ribosomes. RNA helicase has been shown to be related to thermo-tolerance in rice (Wang et al. 2016) and cold tolerance in glacial psychrophilic *Flavobacterium* (Liu et al. 2021). Besides, the tRNA methyltransferase gene interacted with RNA helicase (Fig. 3) has also been verified to be related with temperature changes in bacteria and yeast (Lorenz et al. 2017, Hori 2014). In LFF *Cladonia grayi*, rRNA and the biogenesis of ribosomes have been suggested to be closely related with stress resistance (Armaleo et al. 2019). Our results provide further evidence of the importance of the RNA helicase associated protein network in resistance.

In this study, we chose to perform an extended analysis of regulator of the G-protein signaling (RGS) in another protein network, because G-protein and RGS belong to important pathways that receive external signals in fungi (Lengeler et al. 2000; Zhong and Neubig 2001; McCudden et al. 2005). Orthologous genes of *UaRGS1* involves in the regulation of growth, sporulation, and pathogenicity in model fungi (Chan and Otte 1982; Yu et al. 1996; Ballon et al. 2006; Liu et al. 2007), but there is no experimental evidence that it is related to external low-temperature stimuli or other environmental stress. Here, we verified the new function of RGS related to the ultra-low temperature tolerance. Furthermore, it is worth mentioning that there is a casein kinase I gene in the UaRgs1 protein interaction network (Fig. 3). Casein kinase I is conserved from plants to animals, it performed a number of cellular processes, including DNA repair, cell cycle, cytokinesis, vesicular trafficking, and circadian rhythm (Park et al. 2012; Marzoll et al. 2022). The protein interaction network analysis allows further enrichment of PSGs and can shed more light on the cellular processes in which environmentally adapted genes are involved. The identification of RGS and casein kinase I interaction network in PSGs is more indicative of G-protein signaling pathways that were subjected to selective pressures. Both RGS and casein kinase I are important factors upstream of cellular signaling pathways, and in the case of RGS, as a negative regulator of G-proteins, it is responsible for making the cell to respond correctly to a variety of signals, not just adaptation to cold stress, and it is possible that it will have similar results across different external stresses.

Besides, there is also some other genomic signatures suggesting that the LFF genome is under adaptive selection. The assembly size of *U. aurantiacoatra* is two times as large as those of other relative lichen species, which is caused by LTR amplification and unknown repeat sequences. The LTR amplification is one of the main drivers leading to the big size of eukaryotic genome (Liu et al. 2019b). LTRs show considerable enrichment in lncRNA transcripts compared with non-LTR elements in mouse and human (Kannan et al. 2015; Kapusta et al. 2013; Kelley and Rinn 2012). Most of LTRs transcribed in lncRNAs serve as exons (Kannan et al. 2015), specific families have been co-opted as promoters (Thompson et al. 2016). In plants such as mangrove trees (Lyu et al. 2018), palm (Schley et al. 2022) and bamboo (Papolu et al. 2021), LTR copy number has been reported to crucially associated with adaptation to various environmental stress including aridity or heat. Plenty of evidence suggests that LTR in Plants involves in the modulation of gene expression in response to several stimuli, notably stresses and external challenges (Bui and Grandbastien 2012). In *Medicago sativa*, LTR has been reported to be related to cold tolerance. Study of Sicilian blood oranges *(Citrus sinensis*) illustrated the strength of the LTR as a promoter and as an upstream activating sequence is of cold dependency (Butelli et al. 2012). Therefore, it is reasonable to presume that in the *U. aurantiacoatra* as the symbiont of lichen-forming fungus and plant (alga), the existence of abundant LTR is related to the tolerance of cold environment.

## CONCLUSIONS

In summary, based on our genomic sequencing and bioinformatic analyses, we exhibited several potential mechanisms on how *U. aurantiacoatra* achieved its adaptation to Antarctic environments. We also performed functional validation of one of the genes that has not yet been reported to be associated with stress, further determining the accuracy of the screened PSGs. This study can serve as a good start to understand the environmental adaptions of lichens, and provide the basis and clues for further studies on LFF resistance mechanisms.

## Supplementary Information

Supplementary Material 1: Figure S1. Gene replacement of *UmRGS1*. **a** Diagram of the *UmRGS1* gene and primers used to generated the Δ*Umrgs1* mutant. HY and YG are fragments of the *hph* cassette conferring resistance to hygromycin. **b** Mutant detected with four pairs of anchor primers. Figure S2. GC content-sequencing depth distribution of three usneoid lichen-forming fungi. **a** The left and right panels represent the results of *U. aurantiacoatra* (NJ115-6) using Illumina and Nanopore sequencing platforms, respectively. **b** GC-depth of Illumina-sequencing *U*. sp. (SC-4). **c** GC-depth of Illumina-sequencing *D. longissima* (SC-9). Figure S3. Integrity and characterization of *U. aurantiacoatra* genome. **a** BUSCO assessment of three usneoid LFF genomes with the highest completeness of *U. aurantiacoatra*. **b** Gene feature distribution of *U. aurantiacoatra*. Figure S4. The differences between ω and p values of two strategies. **a** Comparison of ω values of two strategies. **b** Comparison of p values of two strategies.

Supplementary Material 1: Table S1. Information on lichen samples used in this study. Table S2. PCR primers used in this study. Table S3. List of positively selected genes and annotation in Antarctic *Usnea aurantiacoatra*. Table S4. Results of branch site analysis for strategy 1. Table S5. Results of branch site analysis for strategy 2. Table S6. Results of positively selected genes enrichment in GO ontology and KEGG pathway in Antarctic *Usnea aurantiacoatra*. Table S7. Protein interaction networks and annotation of positively selected genes in Antarctic *Usnea aurantiacoatra*. Table S8. Survival colonies and survival rates of WT and the Δ*Umrgs1* mutant after ultra-low-temperature shock.

## Data Availability

The whole genome sequence data of *U. aurantiacoatra* (NJ115-6) have been deposited in the Genome Warehouse in National Genomics Data Center, Beijing Institute of Genomics, Chinese Academy of Sciences/China National Center for Bioinformation, under accession number GWHBJEF00000000 that is publicly accessible at https://ngdc.cncb.ac.cn/gwh. The raw sequence data of *Usnea* sp. (SC-4) and *D. longissima* (SC-9) have been deposited in the Genome Sequence Archive in National Genomics Data Center, China National Center for Bioinformation/Beijing Institute of Genomics, Chinese Academy of Sciences (GSA: CRA007127) that are publicly accessible at https://ngdc.cncb.ac.cn/gsa. The sequence of *UmRGS1* is available in the GenBank Nucleotide Database under accession number ON649705.

## Abbreviations

PSG: Positively selected gene
RGS: Regulator of the G-protein signaling
LFF: Lichen-forming fungi
PAML: Phylogenetic analysis by maximum likelihood
LTR: Long terminal repeat
FDR: False discovery rate

## Declarations

### Adherence to national and international regulations

The authors confirm that this manuscript respects Plant Health and Quarantine and the Nagoya Protocol to the Convention on Biological Diversity.

## References

[CR1] Allen JL, Jones SJM, McMullin RT (2021) Draft genome sequence of the lichenized fungus *Bacidia gigantensis*. Microbiol Resour Announc 10:e0068621 34734769 10.1128/MRA.00686-21 PMC8567793

[CR2] Armaleo D, Müller O, Lutzoni F, Andrésson ÓS, Blanc G, Bode HB, Collart FR, Dal Grande F, Dietrich F, Grigoriev IV, Joneson S, Kuo A, Larsen PE, Logsdon JM, Lopez D, Martin F, May SP, Mcdonald TR, Merchant SS, Miao V, Morin E, Oono R, Pellegrini M, Rubinstein N, Sanchez-Puerta MV, Savelkoul E, Schmitt I, Slot JC, Soanes D, Szovenyi P, Talbot NJ, Veneault-Fourrey C, Xavier BB (2019) The lichen symbiosis re-viewed through the genomes of *Cladonia grayi* and its algal partner *Asterochloris glomerata*. BMC Genomics 20:605–637 31337355 10.1186/s12864-019-5629-x PMC6652019

[CR3] Astashyn A, Tvedte ES, Sweeney D, Sapojnikov V, Bouk N, Joukov V, Mozes E, Strope PK, Sylla PM, Wagner L, Bidwell SL, Brown LC, Clark K, Davis EW, Smith-White B, Hlavina W, Pruitt KD, Schneider VA, Murphy TD (2024) Rapid and sensitive detection of genome contamination at scale with FCS-GX. Genome Biol 25:60 38409096 10.1186/s13059-024-03198-7 PMC10898089

[CR4] Bairoch A, Apweiler R, Wu CH, Barker WC, Boeckmann B, Ferro S, Gasteiger E, Huang HZ, Lopez R, Magrane M, Martin MJ, Natale DA, O’Donovan C, Redaschi N, Yeh LSL (2005) The universal protein resource (UniProt). Nucleic Acids Res 33:D154–D159 15608167 10.1093/nar/gki070 PMC540024

[CR5] Ballon DR, Flanary PL, Gladue DP, Konopka JB, Dohlman HG, Thorner J (2006) DEP-domain-mediated regulation of GPCR signaling responses. Cell 126:1079–1093 16990133 10.1016/j.cell.2006.07.030

[CR6] Benson G (1999) Tandem repeats finder: a program to analyze DNA sequences. Nucleic Acids Res 27:573–580 9862982 10.1093/nar/27.2.573 PMC148217

[CR7] Birney E, Clamp M, Durbin R (2004) GeneWise and genomewise. Genome Res 14:988–995 15123596 10.1101/gr.1865504 PMC479130

[CR8] Bui QT, Grandbastien M-A (2012) LTR retrotransposons as controlling elements of genome response to stress? In: Grandbastien M-A, Casacuberta JM (eds) Plant transposable elements: impact on genome structure and function. Springer, Berlin, Heidelberg

[CR100] Burton JN, Adey A, Patwardhan RP, Qiu R, Kitzman JO, Shendure J (2013) Chromosome-scale scaffolding of de novo genome assemblies based on chromatin interactions. Nat Biotechnol 31:1119–1125 24185095 10.1038/nbt.2727 PMC4117202

[CR9] Burton-Johnson A, Black M, Fretwell PT, Kaluza-Gilbert J (2016) An automated methodology for differentiating rock from snow, clouds and sea in Antarctica from Landsat 8 imagery: a new rock outcrop map and area estimation for the entire Antarctic continent. Cryosphere 10:1665–1677 10.5194/tc-10-1665-2016

[CR10] Butelli E, Licciardello C, Zhang Y, Liu J, Mackay S, Bailey P, Reforgiato-Recupero G, Martin C (2012) Retrotransposons control fruit-specific, cold-dependent accumulation of anthocyanins in blood oranges. Plant Cell 24:1242–1255 22427337 10.1105/tpc.111.095232 PMC3336134

[CR11] Chan RK, Otte CA (1982) Isolation and genetic analysis of *Saccharomyces cerevisiae* mutants supersensitive to G1 arrest by a factor and alpha factor pheromones. Mol Cell Biol 2:11–20 7050665 10.1128/mcb.2.1.11-20.1982 PMC369748

[CR12] Chen N (2004) Using RepeatMasker to identify repetitive elements in genomic sequences. Curr Protoc Bioinform 5:4–10 10.1002/0471250953.bi0410s05 18428725

[CR13] Chen L, Qiu Q, Jiang Y, Wang K, Lin Z, Li Z, Bibi F, Yang Y, Wang J, Nie W, Su W, Liu G, Li Q, Fu W, Pan X, Liu C, Yang J, Zhang C, Yin Y, Wang Y, Zhao Y, Zhang C, Wang Z, Qin Y, Liu W, Wang B, Ren Y, Zhang R, Zeng Y, da Fonseca RR, Wei B, Li R, Wan W, Zhao R, Zhu W, Wang Y, Duan S, Gao Y, Zhang YE, Chen C, Hvilsom C, Epps CW, Chemnick LG, Dong Y, Mirarab S, Siegismund HR, Ryder OA, Gilbert MTP, Lewin HA, Zhang G, Heller R, Wang W (2019) Large-scale ruminant genome sequencing provides insights into their evolution and distinct traits. Science 364:eaav6202 31221828 10.1126/science.aav6202

[CR14] Chin CS, Alexander DH, Marks P, Klammer AA, Drake J, Heiner C, Clum A, Copeland A, Huddleston J, Eichler EE, Turner SW, Korlach J (2013) Nonhybrid, finished microbial genome assemblies from long-read SMRT sequencing data. Nat Methods 10:563–569 23644548 10.1038/nmeth.2474

[CR15] Daane JM, Detrich HW (2022) Adaptations and diversity of Antarctic fishes: a genomic perspective. Ann Rev Anim Biosci 10:39–62 34748709 10.1146/annurev-animal-081221-064325

[CR16] Divakar PK, Crespo A, Kraichak E, Leavitt SD, Singh G, Schmitt I, Lumbsch HT (2017) Using a temporal phylogenetic method to harmonize family-and genus-level classification in the largest clade of lichen-forming fungi. Fungal Divers 84:101–117 10.1007/s13225-017-0379-z

[CR17] Esseen P-A, Ericson L, Lindström H, Zackrisson O (1981) Occurrence and ecology of usnea longissima in central Sweden. Lichenologist 13:177–190 10.1017/S0024282981000224

[CR18] Flynn JM, Hubley R, Goubert C, Rosen J, Clark AG, Feschotte C, Smit AF (2020) RepeatModeler2 for automated genomic discovery of transposable element families. Proc Natl Acad Sci USA 117:9451–9457 32300014 10.1073/pnas.1921046117 PMC7196820

[CR19] Griffiths-Jones S, Moxon S, Marshall M, Khanna A, Eddy SR, Bateman A (2005) Rfam: annotating non-coding RNAs in complete genomes. Nucleic Acids Res 33:D121–D124 15608160 10.1093/nar/gki081 PMC540035

[CR20] Grigoriev IV, Nikitin R, Haridas S, Kuo A, Ohm R, Otillar R, Riley R, Salamov A, Zhao XL, Korzeniewski F, Smirnova T, Nordberg H, Dubchak I, Shabalov I (2014) MycoCosm portal: gearing up for 1000 fungal genomes. Nucleic Acids Res 42:D699–D704 24297253 10.1093/nar/gkt1183 PMC3965089

[CR21] Haas BJ, Salzberg SL, Zhu W, Pertea M, Allen JE, Orvis J, White O, Buell CR, Wortman JR (2008) Automated eukaryotic gene structure annotation using EVidenceModeler and the program to assemble spliced alignments. Genome Biol 9:R7 18190707 10.1186/gb-2008-9-1-r7 PMC2395244

[CR22] Hawksworth DL, Grube M (2020) Lichens redefined as complex ecosystems. New Phytol 227:1281–1283 32484275 10.1111/nph.16630 PMC7497170

[CR23] Hecker N, Hiller M (2020) A genome alignment of 120 mammals highlights ultraconserved element variability and placenta-associated enhancers. GigaScience 9:giz159 31899510 10.1093/gigascience/giz159 PMC6941714

[CR24] Honegger R (1991) Symbiosis and fungal evolution: symbiosis and morphogenesis. In: Margulis L, Fester R (eds) Evolution and speciation: symbiosis as a source of evolutionary innovation. MIT Press 11538111

[CR25] Hori H (2014) Methylated nucleosides in tRNA and tRNA methyltransferases. Front Genet 5:144 24904644 10.3389/fgene.2014.00144 PMC4033218

[CR26] Jansson KU, Palmqvist K, Esseen P-A (2009) Growth of the old forest lichen *Usnea longissima* at forest edges. Lichenologist 41:663–672 10.1017/S0024282909008536

[CR27] Kannan S, Chernikova D, Rogozin IB, Poliakov E, Managadze D, Koonin EV, Milanesi L (2015) Transposable element insertions in long intergenic non-coding RNA genes. Front in Bioeng Biotechnol 3:71 10.3389/fbioe.2015.00071 PMC4460805 26106594

[CR28] Kapusta A, Kronenberg Z, Lynch VJ, Zhuo X, Ramsay L, Bourque G, Yandell M, Feschotte C (2013) Transposable elements are major contributors to the origin, diversification, and regulation of vertebrate long noncoding RNAs. PLoS Genet 9:e1003470 23637635 10.1371/journal.pgen.1003470 PMC3636048

[CR29] Keilwagen J, Wenk M, Erickson JL, Schattat MH, Grau J, Hartung F (2016) Using intron position conservation for homology-based gene prediction. Nucleic Acids Res 44:e89 26893356 10.1093/nar/gkw092 PMC4872089

[CR30] Kelley D, Rinn J (2012) Transposable elements reveal a stem cell-specific class of long noncoding RNAs. Genome Biol 13:R107 23181609 10.1186/gb-2012-13-11-r107 PMC3580499

[CR31] Keon D (2009) Growth of *Usnea longissima* across a variety of habitats in the oregon coast range. Bryologist 105:233–242 10.1639/0007-2745(2002)105[0233:GOULAA]2.0.CO;2

[CR32] Klionsky DJ, Herman PK, Emr SD (1990) The fungal vacuole-composition, function, and biogenesis. Microbiol Rev 54:266–292 2215422 10.1128/mr.54.3.266-292.1990 PMC372777

[CR33] Krzywinski M, Schein J, Birol I, Connors J, Gascoyne R, Horsman D, Jones SJ, Marra MA (2009) Circos: an information aesthetic for comparative genomics. Genome Res 19:1639–1645 19541911 10.1101/gr.092759.109 PMC2752132

[CR34] Lagesen K, Hallin P, Rodland EA, Stærfeldt HH, Rognes T, Ussery DW (2007) RNAmmer: consistent and rapid annotation of ribosomal RNA genes. Nucleic Acids Res 35:3100–3108 17452365 10.1093/nar/gkm160 PMC1888812

[CR35] Langmead B, Salzberg SL (2012) Fast gapped-read alignment with Bowtie 2. Nat Methods 9:357-U54 22388286 10.1038/nmeth.1923 PMC3322381

[CR36] Lengeler KB, Davidson RC, D’Souza C, Harashima T, Shen WC, Wang P, Pan XW, Waugh M, Heitman J (2000) Signal transduction cascades regulating fungal development and virulence. Microbiol Mol Biol Rev 64:746–785 11104818 10.1128/MMBR.64.4.746-785.2000 PMC99013

[CR37] Li C, Zhang Y, Li JW, Kong LS, Hu HF, Pan HL, Xu LH, Deng Y, Li QY, Jin LJ, Yu H, Chen Y, Liu BH, Yang LF, Liu SP, Zhang Y, Lang YS, Xia JQ, He WM, Shi Q, Subramanian S, Millar CD, Meader S, Rands CM, Fujita MK, Greenwold MJ, Castoe TA, Pollock D, Gu WJ, Nam K, Ellegren H, Ho SYW, Burt DW, Ponting CP, Jarvis ED, Gilbert MTP, Yang HM, Wang J, Lambert DM, Wang J, Zhang GJ (2014a) Two Antarctic penguin genomes reveal insights into their evolutionary history and molecular changes related to the Antarctic environment. Gigascience 3:1–15 25671092 10.1186/2047-217X-3-27 PMC4322438

[CR38] Li Y, Kromer B, Schukraft G, Bubenzer O, Huang M-R, Wang Z-M, Bian L-G, Li C-S (2014b) Growth rate of *Usnea aurantiacoatra* (Jacq.) Bory on Fildes Peninsula, Antarctica and its climatic background. PLoS ONE 9:e100735 24968131 10.1371/journal.pone.0100735 PMC4072682

[CR39] Liu H, Suresh A, Willard FS, Siderovski DP, Lu S, Naqvi NI (2007) Rgs1 regulates multiple Gα subunits in *Magnaporthe* pathogenesis, asexual growth and thigmotropism. EMBO J 26:690–700 17255942 10.1038/sj.emboj.7601536 PMC1794393

[CR40] Liu FF, Chen SF, Ferreira MA, Chang RL, Sayari M, Kanzi AM, Wingfield BD, Wingfield MJ, Pizarro D, Crespo A, Divakar PK, de Beer ZW, Duong TA (2019a) Draft genome sequences of five *Calonectria* species from *Eucalyptus* plantations in China, *Celoporthe dispersa*, *Sporothrix phasma* and *Alectoria sarmentosa*. IMA Fungus 10:1–13 32647626 10.1186/s43008-019-0023-5 PMC7325655

[CR41] Liu Y, Ul Qamar MT, Feng JW, Ding YD, Wang S, Wu GZ, Ke LJ, Xu Q, Chen LL (2019b) Comparative analysis of miniature inverted-repeat transposable elements (MITEs) and long terminal repeat (LTR) retrotransposons in six *Citrus* species. BMC Plant Biol 19:1–16 30987586 10.1186/s12870-019-1757-3 PMC6466647

[CR42] Liu Q, Li W, Liu D, Li LY, Li J, Lv N, Liu F, Zhu BL, Zhou YG, Xin YH, Dong XZ (2021) Light stimulates anoxic and oligotrophic growth of glacial Flavobacterium strains that produce zeaxanthin. ISME J 15:1844–1857 33452478 10.1038/s41396-020-00891-w PMC8163750

[CR43] Lorenz C, Lünse CE, Mörl M (2017) tRNA modifications: impact on structure and thermal adaptation. Biomolecules 7:35 28375166 10.3390/biom7020035 PMC5485724

[CR44] Lowe TM, Eddy SR (1997) tRNAscan-SE: a program for improved detection of transfer RNA genes in genomic sequence. Nucleic Acids Res 25:955–964 9023104 10.1093/nar/25.5.955 PMC146525

[CR45] Lücking R, Nelsen MP (2018) Ediacarans, Protolichens, and lichen-derived *Penicillium*: a critical reassessment of the evolution of lichenization in fungi. In: Krings M, Harper CJ, Cúneo NR, Rothwell GW (eds) Transformative paleobotany. Academic Press

[CR46] Lyu HM, He ZW, Wu CI, Shi SH (2018) Convergent adaptive evolution in marginal environments: unloading transposable elements as a common strategy among mangrove genomes. New Phytol 217:428–438 28960318 10.1111/nph.14784

[CR47] Marzoll D, Serrano FE, Shostak A, Schunke C, Diernfellner ACR, Brunner M (2022) Casein kinase 1 and disordered clock proteins form functionally equivalent, phospho-based circadian modules in fungi and mammals. Proc Natl Acad Sci USA 119:e2118286119 35217617 10.1073/pnas.2118286119 PMC8892514

[CR48] McCudden CR, Hains MD, Kimple RJ, Siderovski DP, Willard FS (2005) G-protein signaling: back to the future. Cell Mol Life Sci 62:551–577 15747061 10.1007/s00018-004-4462-3 PMC2794341

[CR49] McKenzie SK, Walston RF, Allen JL (2020) Complete, high-quality genomes from long-read metagenomic sequencing of two wolf lichen thalli reveals enigmatic genome architecture. Genomics 112:3150–3156 32504651 10.1016/j.ygeno.2020.06.006

[CR50] Meiser A, Otte J, Schmitt I, dal Grande F (2017) Sequencing genomes from mixed DNA samples—evaluating the metagenome skimming approach in lichenized fungi. Sci Rep 7:14881 29097759 10.1038/s41598-017-14576-6 PMC5668418

[CR51] Merges D, dal Grande F, Valim H, Singh G, Schmitt I (2023) Gene abundance linked to climate zone: parallel evolution of gene content along elevation gradients in lichenized fungi. Front Microbiol 14:1097787 37032854 10.3389/fmicb.2023.1097787 PMC10073550

[CR52] O’Day CL, Chavanikamannil F, Abelson J (1996) 18S rRNA processing requires the RNA helicase-like protein Rrp3. Nucleic Acids Res 24:3201–3207 8774901 10.1093/nar/24.16.3201 PMC146083

[CR53] Ogata H, Goto S, Sato K, Fujibuchi W, Bono H, Kanehisa M (1999) KEGG: Kyoto encyclopedia of genes and genomes. Nucleic Acids Res 27:29–34 9847135 10.1093/nar/27.1.29 PMC148090

[CR54] Øvstedal DO, Smith RL (2001) Lichens of Antarctica and South Georgia: a guide to their identification and ecology. Cambridge University Press

[CR55] Papolu PK, Ramakrishnan M, Wei Q, Vinod KK, Zou LH, Yrjala K, Kalendar R, Zhou MB (2021) Long terminal repeats (LTR) and transcription factors regulate *PHRE1* and *PHRE2* activity in Moso bamboo under heat stress. BMC Plant Biol 21:585 34886797 10.1186/s12870-021-03339-1 PMC8656106

[CR56] Park YI, Do KH, Kim IS, Park HH (2012) Structural and functional studies of casein kinase I-like protein from rice. Plant Cell Physiol 53:304–311 22199373 10.1093/pcp/pcr175

[CR57] Parra G, Blanco E, Guigó R (2000) GeneID in Drosophila. Genome Res 10:511–515 10779490 10.1101/gr.10.4.511 PMC310871

[CR58] Remm M, Storm CEV, Sonnhammer ELL (2001) Automatic clustering of orthologs and in-paralogs from pairwise species comparisons. J Mol Biol 314:1041–1052 11743721 10.1006/jmbi.2000.5197

[CR59] Romeike J, Friedl T, Helms G, Ott S (2002) Genetic diversity of algal and fungal partners in four species of Umbilicaria (Lichenized ascomycetes) along a transect of the Antarctic Peninsula. Mol Biol Evol 19:1209–1217 12140232 10.1093/oxfordjournals.molbev.a004181

[CR60] Sancho LG, Pintado A, Navarro F, Ramos M, de Pablo MA, Blanquer JM, Raggio J, Valladares F, Green TGA (2017) Recent warming and cooling in the Antarctic Peninsula Region has rapid and large effects on lichen vegetation. Sci Rep 7:5689 28740147 10.1038/s41598-017-05989-4 PMC5524963

[CR61] Schley RJ, Pellicer J, Ge XJ, Barrett C, Bellot S, Guignard MS, Novak P, Suda J, Fraser D, Baker WJ, Dodsworth S, Macas J, Leitch AR, Leitch IJ (2022) The ecology of palm genomes: repeat-associated genome size expansion is constrained by aridity. New Phytol 236:433–446 35717562 10.1111/nph.18323 PMC9796251

[CR62] Servant N, Varoquaux N, Lajoie BR, Viara E, Chen CJ, Vert JP, Heard E, Dekker J, Barillot E (2015) HiC-Pro: an optimized and flexible pipeline for Hi-C data processing. Genome Biol 16:1–11 26619908 10.1186/s13059-015-0831-x PMC4665391

[CR63] Singh G, Calchera A, Schulz M, Drechsler M, Bode HB, Schmitt I, dal Grande F (2021) Climate-specific biosynthetic gene clusters in populations of a lichen-forming fungus. Environ Microbiol 23:4260–4275 34097344 10.1111/1462-2920.15605

[CR64] Son Y-E, Jung WH, Oh S-H, Kwak J-H, Cárdenas ME, Park H-S (2018) Mon1 is essential for fungal virulence and stress survival in *Cryptococcus neoformans*. Mycobiology 46:114–121 29963312 10.1080/12298093.2018.1468053 PMC6023253

[CR65] Stanke M, Schöffmann O, Morgenstern B, Waack S (2006) Gene prediction in eukaryotes with a generalized hidden Markov model that uses hints from external sources. BMC Bioinform 7:1–11 10.1186/1471-2105-7-62 PMC1409804 16469098

[CR66] Thell A, Crespo A, Divakar PK, Kärnefelt I, Leavitt SD, Lumbsch HT, Seaward MR (2012) A review of the lichen family Parmeliaceae–history, phylogeny and current taxonomy. Nordic J Bot 30:641–664 10.1111/j.1756-1051.2012.00008.x

[CR67] Thompson PJ, Macfarlan TS, Lorincz MC (2016) Long terminal repeats: from parasitic elements to building blocks of the transcriptional regulatory repertoire. Mol Cell 62:766–776 27259207 10.1016/j.molcel.2016.03.029 PMC4910160

[CR68] Walker BJ, Abeel T, Shea T, Priest M, Abouelliel A, Sakthikumar S, Cuomo CA, Zeng QD, Wortman J, Young SK, Earl AM (2014) Pilon: an integrated tool for comprehensive microbial variant detection and genome assembly improvement. PLoS ONE 9:e112963 25409509 10.1371/journal.pone.0112963 PMC4237348

[CR69] Wang D, Qin BX, Li X, Tang D, Zhang Y, Cheng ZK, Xue YB (2016) Nucleolar DEAD-Box RNA helicase TOGR1 regulates thermotolerant growth as a pre-rRNA chaperone in rice. Plos Genet 12:e1005844 26848586 10.1371/journal.pgen.1005844 PMC4743921

[CR70] Wang Y, Wei X, Bian Z, Wei J, Xu JR (2020) Coregulation of dimorphism and symbiosis by cyclic AMP signaling in the lichenized fungus *Umbilicaria muhlenbergii*. Proc Natl Acad Sci USA 117:23847–23858 32873646 10.1073/pnas.2005109117 PMC7519320

[CR71] Wang Y, Li R, Wang D, Qian B, Bian Z, Wei J, Wei X, Xu J-R (2023) Regulation of symbiotic interactions and primitive lichen differentiation by *UMP1* MAP kinase in *Umbilicaria muhlenbergii*. Nat Commun 14:6972 37914724 10.1038/s41467-023-42675-8 PMC10620189

[CR72] Wauchope HS, Shaw JD, Terauds A (2019) A snapshot of biodiversity protection in Antarctica. Nat Commun 10:946 30808907 10.1038/s41467-019-08915-6 PMC6391489

[CR85] White TJ, Bruns T, Lee S, Taylor J (1990) Amplification and direct sequencing of fungal ribosomal RNA genes for phylogenetics. In: Innis MA, Gelfand DH, Sninsky JJ, White TJ (eds) PCR Protocols. Academic Press

[CR73] Wilken PM, Aylward J, Chand R, Grewe F, Lane FA, Sinha S, Ametrano C, Distefano I, Divakar PK, Duong TA, Huhndorf S, Kharwar RN, Lumbsch HT, Navathe S, Pérez CA, Ramírez-Berrutti N, Sharma R, Sun YK, Wingfield BD, Wingfield MJ (2020) IMA genome-F13: draft genome sequences of Ambrosiella cleistominuta, *Cercospora brassicicola*, *C. citrullina*, *Physcia stellaris*, and *Teratosphaeria pseudoeucalypti*. IMA Fungus 11:1–17 33014691 10.1186/s43008-020-00039-7 PMC7513301

[CR74] Wirtz N, Printzen C, Sancho LG, Lumbsch TH (2006) The phylogeny and classification of Neuropogon and Usnea (Parmeliaceae, Ascomycota) revisited. Taxon 55:367–376 10.2307/25065584

[CR75] Wu T, Hu E, Xu S, Chen M, Guo P, Dai Z, Feng T, Zhou L, Tang W, Zhan L, Fu X, Liu S, Bo X, Yu G (2021) clusterProfiler 4.0: a universal enrichment tool for interpreting omics data. Innovation (camb) 2:100141 34557778 10.1016/j.xinn.2021.100141 PMC8454663

[CR76] Yang Z (2007) PAML 4: phylogenetic analysis by maximum likelihood. Mol Biol Evol 24(8):1586–1591 17483113 10.1093/molbev/msm088

[CR77] Yang QX, Wang YY, Lücking R, Lumbsch HT, Du ZY, Chen YK, Bai M, Ren D, Wei JC, Li H, Wang YJ, Wei XL (2023) The Jurassic epiphytic macrolichen reveals the oldest lichen-plant interaction in a Mesozoic forest ecosystem. iScience 26:105770 36590161 10.1016/j.isci.2022.105770 PMC9800524

[CR78] Yu JH, Wieser J, Adams TH (1996) The *Aspergillus* FlbA RGS domain protein antagonizes G protein signaling to block proliferation and allow development. EMBO J 15:5184–5190 8895563 10.1002/j.1460-2075.1996.tb00903.x PMC452262

[CR79] Zdobnov EM, Apweiler R (2001) InterProScan - an integration platform for the signature-recognition methods in InterPro. Bioinformatics 17:847–848 11590104 10.1093/bioinformatics/17.9.847

[CR80] Zhang ZH, Qu CF, Zhang KJ, He YY, Zhao X, Yang LX, Zheng Z, Ma XY, Wang XX, Wang WY, Wang K, Li D, Zhang LP, Zhang X, Su DY, Chang X, Zhou MY, Gao D, Jiang WK, Leliaert F, Bhattacharya D, de Clerck O, Zhong BJ, Miao JL (2020) Adaptation to extreme Antarctic environments revealed by the genome of a sea ice green alga. Curr Biol 30:3330–3341 32619486 10.1016/j.cub.2020.06.029

[CR81] Zhang TT, Grube M, Wei XL (2023) Host selection tendency of key microbiota in arid desert lichen crusts. iMeta e138 10.1002/imt2.138 PMC10989926 38868215

[CR82] Zhao X, Kim Y, Park G, Xu J-R (2005) A mitogen-activated protein kinase cascade regulating infection-related morphogenesis in *Magnaporthe grisea*. Plant Cell 17:1317–1329 15749760 10.1105/tpc.104.029116 PMC1088005

[CR83] Zhong HL, Neubig RR (2001) Regulator of G protein signaling proteins: novel multifunctional drug targets. J Pharmacol Exp Ther 297:837–845 11356902

[CR84] Zhu Y, Zhou D, Bai NS, Liu Q, Zhao N, Yang J (2023) SNARE protein AoSec22 orchestrates mycelial growth, vacuole assembly, trap formation, stress response, and secondary metabolism in *Arthrobotrys oligospora*. J Fungi 9:75–91 10.3390/jof9010075 PMC9863257 36675896

