# Supplementary material for: Exploration on cold adaptation of Antarctic lichen via detection of positive selection genes
Source: IMA Fungus. 2024 Sep 9;15:29. doi: 10.1186/s43008-024-00160-x (PMC11386357; doi:10.1186/s43008-024-00160-x)
Supplement: Supplementary file 1 — Supplementary Material 1: Figure S1. Gene replacement of UmRGS1. a Diagram of the UmRGS1 gene and primers used to generated the ΔUmrgs1 mutant. HY and YG are fragments of the hph cassette conferring resistance to hygromycin. b Mutant detected with four pairs of anchor primers. Figure S2. GC content-sequencing depth distribution of three usneoid lichen-forming fungi. a The left and right panels represent the results of U. aurantiacoatra (NJ115-6) using Illumina and Nanopore sequencing platforms, respectively. b GC-depth of Illumina-sequencing U. sp. (SC-4). c GC-depth of Illumina-sequencing D. longissima (SC-9). Figure S3. Integrity and characterization of U. aurantiacoatra genome. a BUSCO assessment of three usneoid LFF genomes with the highest completeness of U. aurantiacoatra. b Gene feature distribution of U. aurantiacoatra. Figure S4. The differences between ω and p values of two strategies. a Comparison of ω values of two strategies. b Comparison of p values of two strategies. [file 43008_2024_160_MOESM1_ESM.docx]

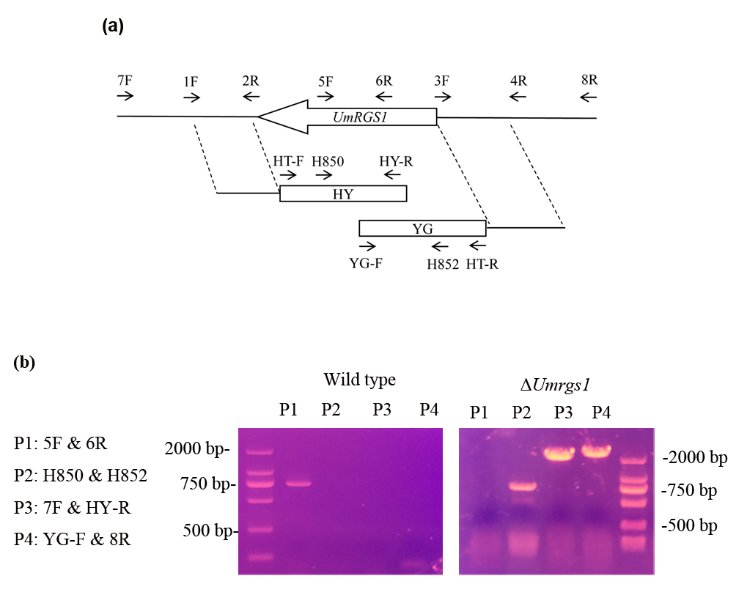


**Figure S1. Gene replacement of *UmRGS1*. (a)** Diagram of the *UmRGS1* gene and primers used to generated the Δ*Umrgs1* mutant. HY and YG are fragments of the *hph* cassette conferring resistance to hygromycin. **(b)** Mutant detected with four pairs of anchor primers.


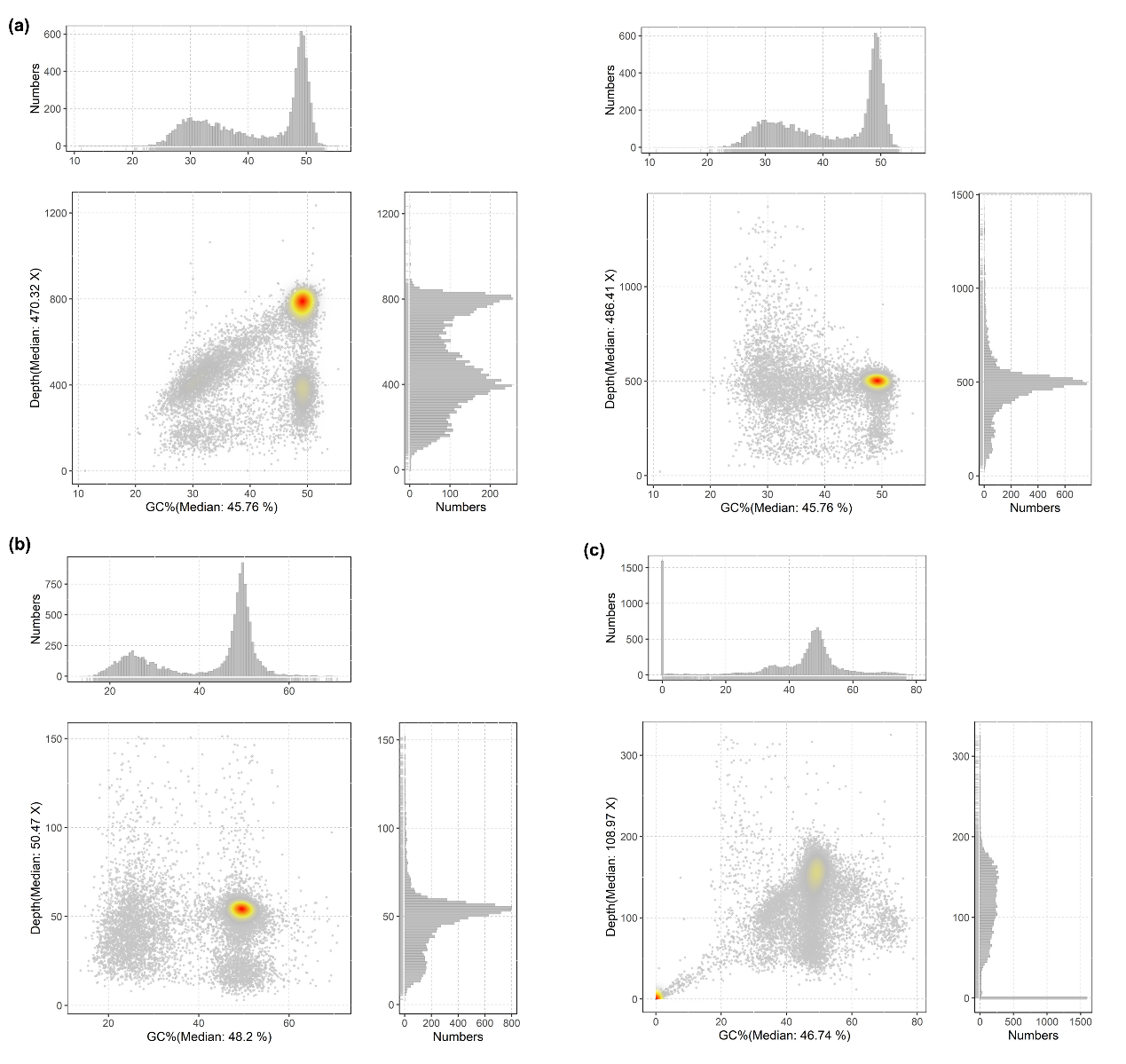


**Figure S2. GC content-sequencing depth distribution of three usneoid lichen-forming fungi.** **(a)** The left and right panels represent the results of *U. aurantiacoatra* (NJ115-6) using Illumina and Nanopore sequencing platforms, respectively. **(b)** GC-depth of Illumina-sequencing *U.* sp. (SC-4). **(c)** GC-depth of Illumina-sequencing *D. longissima* (SC-9).


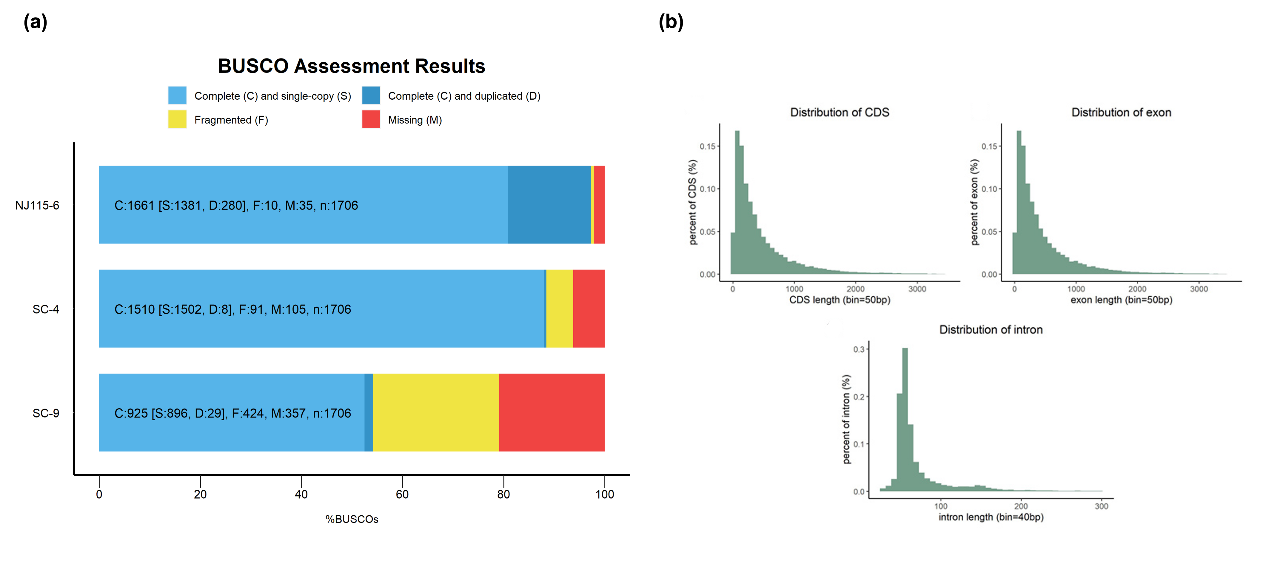


**Figure S3. Integrity and characterization of *U. aurantiacoatra* genome. (a)** BUSCO assessment of three usneoid LFF genomes with the highest completeness of *U. aurantiacoatra*. **(b)** Gene feature distribution of *U. aurantiacoatra*.


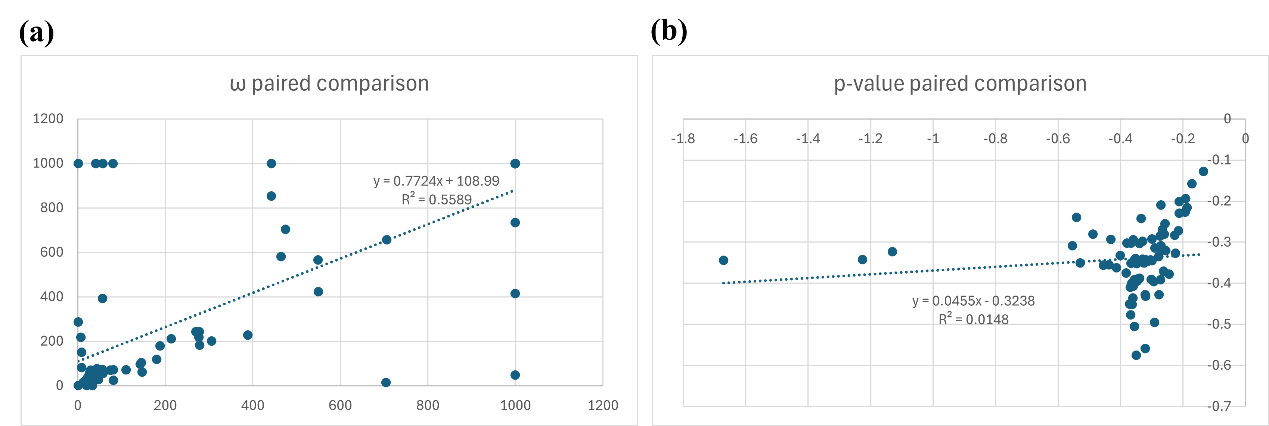


**Figure S4. The differences between** **ω and p values of two strategies.** **(a)** Comparison of ω values of two strategies. **(b)** Comparison of p values of two strategies.
